# Transcatheter aortic valve implantation (from inception to standard treatment): a single-center observational study

**DOI:** 10.3389/fcvm.2024.1298346

**Published:** 2024-01-15

**Authors:** Martin Petter Høydahl, Rolf Busund, Assami Rösner, Didrik Kjønås

**Affiliations:** ^1^ Clinical Cardiovascular Research Group, Institute of Clinical Medicine, The Arctic University of Norway, Tromsø, Norway; ^2^ Department of Cardiothoracic Surgery, University Hospital of North Norway, Tromsø, Norway; ^3^ Department of Cardiology, University Hospital of North Norway, Tromsø, Norway; ^4^ Department of Gastrointestinal Surgery, University Hospital of North Norway, Tromsø, Norway

**Keywords:** transcatheter aortic valve implantation, transcatheter aortic valve replacement, aortic stenosis, mortality, complications

## Abstract

**Background:**

Treatment of severe aortic stenosis with transcatheter aortic valve implantation (TAVI) was introduced in 2002. Since then, TAVI has become the primary treatment approach worldwide for advanced-age patients and younger patients with severe comorbidities. We aimed to evaluate the changes in patient demographics, complications, and mortality rates within 13 years.

**Methods:**

This retrospective observational study included 867 patients who underwent TAVI at the University Hospital of North Norway in Tromsø from 2008 to 2021. The 13-year period was divided into period 1 (2008–2012), period 2 (2013–2017), and period 3 (2018–2021). The primary objective was to evaluate the changes in periprocedural (30 days), early (30–365 days), and late mortality rates (>365 days) between the periods. The secondary objective was to evaluate late mortality rates by sex and age groups: <70 years, 70–79 years, 80–89 years, and ≥90 years.

**Results:**

The periprocedural mortality rates for periods 1, 2, and 3 were 10.3%, 2.9%, and 1.2%, respectively (*P* < 0.001). The early mortality rates were 5.6%, 5.8%, and 6.5%, respectively. No significant differences were observed in late mortality by sex or age group (<70, 70–79, and 80–89 years) with a median survival of 5.3–5.6 years. The median survival in patients aged ≥90 years was 4.0 years (*P* = 0.018).

**Conclusion:**

Our findings indicate that most patients are octogenarians, and the burden of their comorbidities should be highly considered compared to their age when evaluating the procedural outcomes. As the incidence of most complications related to TAVI has decreased, the rates of permanent pacemaker implantation remain high. Important advancements in diagnostics, valve technology, and procedural techniques have improved the periprocedural mortality rates; however, early mortality remains unchanged and poses a clinical challenge that needs to be addressed in the future.

## 1 Introduction

In 2002, a French cardiologist and professor Alain Cribier performed the first transcatheter aortic valve implantation (TAVI) (1). Since then, TAVI has become the primary treatment modality for severe symptomatic aortic stenosis (AS). AS due to degenerative calcification is the predominant valvular heart disease in high-income countries (2), with a projected twofold increase in prevalence within the next five decades (3, 4). Previously, surgical aortic valve replacement (SAVR) was the only curative option prior to the introduction of TAVI. Currently, SAVR is primarily recommended for patients aged <70 years, with bicuspid valve anatomy, with concomitant multivessel coronary artery disease (CAD), with cardiac conditions requiring surgery, or deemed unsuitable for TAVI. In certain cases, patients may opt for a mechanical valve, despite the necessity for lifelong anticoagulation, owing to the lower rates of re-intervention compared with bioprosthetic valves. Previous randomized controlled trials have demonstrated comparable outcomes between TAVI and SAVR, with TAVI showing a favorable trend owing to its less invasive nature (5–12).

Current issues facing TAVI are the long-term durability of transcatheter heart valves (THVs), high rates of permanent pacemaker implantation (PPI), and the long-term impact of mild paravalvular leak (PVL) (13–15). As the indication of TAVI is expanded to younger age groups, the complexity of choosing the right intervention increases (16). Therefore, a multidisciplinary team is fundamental in ensuring that patients receive thorough information to enable effective shared decision-making (17, 18).

As the first university hospital in Norway, the University Hospital of Northern Norway (UNN) in Tromsø introduced its TAVI program in 2008. This study aimed to evaluate the changes in patient demographics, complications, and mortality rates associated with TAVI performed in a single center within a period of 13 years.

## 2 Materials and methods

### 2.1 Patient population and data collection

The study cohort included all patients with symptomatic severe AS who underwent TAVI at UNN Tromsø from September 2008 through December 2021. This 13-year timeframe was further divided into three periods according to the changes in the European guidelines for the treatment of valvular heart disease by the European Society of Cardiology and the European Association for Cardio-Thoracic Surgery in 2012, 2017, and 2021 (19), respectively. The periods were chosen to examine guideline influences on patient selection and outcome, reduce selection bias and improve overall homogeneity. Periprocedural mortality, early mortality, late mortality, and complications were defined according to the Valve Academic Research Consortium 3 (VARC-3) criteria (20). The clinical characteristics, complications, and mortality data of all patients were retrospectively collected from the electronic medical records to obtain comprehensive information. The missing values were not imputed, and none of the patients were lost to follow-up, thus ensuring data integrity and completeness. The local data protection office approved the collection of data.

### 2.2 Preprocedural workup and procedural characteristics

All patients underwent clinical evaluation, transthoracic or transesophageal echocardiography (TEE), and computed tomography (CT) at our center to evaluate the disease severity and feasibility of TAVI. The heart team evaluated the suitability of each patient for TAVI. The contraindications for the procedure were a lifetime expectancy of less than 1 year, inability to provide informed consent, and low motivation, as expressed by the patient. The available version of the European System for Cardiac Operative Risk Evaluation (EuroSCORE) I/log/II was used for risk stratification.

All procedures were performed by a cardiovascular surgeon and interventional cardiologist. At their discretion, either a balloon-expanding valve (BEV) or a self-expanding valve (SEV) was used. The routes of access were as follows: trans-femoral (TF-TAVI), -apical (TA-TAVI), -aortic (TAo-TAVI), -carotid (TC-TAVI), or -subclavian/axillary (TSc-TAVI). The anesthesia used was either general or conscious sedation with localized anesthesia.

### 2.3 Statistical analysis

Continuous variables are presented as the mean ± standard deviation or the median with an interquartile range depending on the normality of distribution. Normally distributed variables were compared using Student's *t*-test or analysis of variance, while non-normally distributed variables were compared using the Wilcoxon rank-sum test. The Shapiro–Wilk test was used to assess the normality of the distribution. The categorical variables were presented as numbers with percentages and compared using the *X*^2^ test or Fisher's exact test if the expected event count was less than 5. Kaplan–Meier plots were used to present the time-to-event analysis, and the log-rank test was used to compare their differences. Potential predictors were assessed for clinical relevance and multicollinearity, and included in a backwards stepwise multivariable logistic regression analysis if the *P*-value ≤ 0.15. A two-tailed *P*-value of <0.05 was considered significant. STATA 17.0 (StataCorp. 2021. Stata Statistical Software: Release 17. College Station, TX: StataCorp LLC.was) was used for all analyses.

## 3 Results

### 3.1 Patients

The baseline characteristics of the 867 patients who underwent TAVI between 2008 and 2021 are presented in Table 1. In general, period 1 exhibited a higher proportion of women and older patients. Additionally, there was a higher prevalence of NYHA class ≥3, previous myocardial infarction and higher mean pre-operative gradients. The estimated EuroSCORE II significantly decreased, transitioning from extreme to high risk in periods 1 and 2 to intermediate to low risk in period 3. From period 2 to period 3 the prevalence of CAD, peripheral artery disease, systemic corticosteroid (SCT) usage, previous coronary artery bypass graft (CABG) surgery and previous cardiac surgery decreased. Across all periods the median aortic-valve area (AVA) increased, but rates of severe pulmonary hypertension declined.

**Table 1:** Baseline characteristics between periods.

	All patients (*N* = 867)	2008–2012 (*N* = 126)	2013–2017 (*N* = 308)	2018–2021 (*N* = 433)	*P*-value
Age, years	82 (77–86)	84 (80–87)	81 (77–86)	82 (77–86)	<0.001*
Male sex	445 (51.3)	51 (40.5)	171 (55.5)	223 (51.5)	0.017*
NYHA class ≥3	769 (88.7)	120 (95.2)	270 (87.7)	379 (87.5)	0.043*
EuroSCORE I	10 (9–12)	12 (10–13)	10 (9–12)	9 (8–11)	<0.001*^,^^†^
Logistic EuroSCORE	18.0 (11.3–29.6)	26.0 (17.2–37.2)	20.4 (13.2–34.4)	14.5 (8.6–22.9)	<0.001*^,^^†^
EuroSCORE II	5.3 (3.2–9.2)	7.6 (4.9–10.5)	6.0 (3.6–10.9)	4.3 (2.7–7.3)	<0.001^†^
Body-mass-index, kg/m^2^	26.0 (23.2–29.3)	25.0 (22.6–29.4)	26.1 (23.5–28.9)	26.2 (23.4–29.4)	0.395
Aortic-valve area, cm^2^	0.6 (0.5–0.7)	0.50 (0.44–0.60)	0.60 (0.50–0.70)	0.64 (0.50–0.86)	<0.001*^,^^†^
Body surface area, m^2^	1.82 (1.67–1.96)	1.75 (1.62–1.89)	1.86 (1.66–1.99)	1.82 (1.68–1.98)	<0.001*
Pre-operative LVEF	54 (45–60)	55 (45–62)	53 (40–60)	55 (45–60)	0.441
Pre-operative gradient	(*N* = 860) 51 (44–60)	(*N* = 125) 58 (49–70)	(*N* = 306) 50 (43–60)	(*N* = 430) 50 (44–58)	<0.001*
eGFR, mean ± SD	63.3 ± 23.2	63.4 ± 22.3	64.2 ± 24.0	65.2 ± 23.0	0.687
Mitral valve regurgitation >2	69 (8.0)	4 (3.2)	42 (13.6)	23 (5.3)	<0.001*^,^^†^
Aortic valve regurgitation >2	93 (10.7)	11 (8.7)	41 (13.3)	41 (9.5)	0.184
Hypertension^a^	536 (61.8)	62 (49.2)	183 (59.4)	291 (67.2)	<0.001^†^
Diabetes mellitus	183 (21.1)	23 (18.3)	66 (21.4)	94 (21.7)	0.694
CAD	502 (57.9)	82 (65.1)	191 (62.0)	229 (52.9)	0.01^†^
Previous MI	253 (29.2)	57 (45.2)	92 (29.9)	104 (24.0)	<0.001*
Cerebrovascular disease	214 (24.7)	24 (19.0)	82 (26.6)	108 (24.9)	0.284
Previous stroke	131 (15.1)	17 (13.5)	50 (16.2)	64 (14.8)	0.742
PAD	253 (29.2)	39 (31.0)	107 (34.7)	107 (24.7)	0.011^†^
COPD	258 (29.8)	36 (28.6)	84 (27.3)	138 (31.9)	0.383
Systemic corticosteroid treatment	159 (18.3)	29 (23.0)	68 (22.1)	62 (14.3)	0.009
Porcelain aorta	24 (2.8)	5 (4.0)	11 (3.6)	8 (1.8)	0.250
Cancer^b^	185 (21.3)	18 (14.3)	69 (22.4)	98 (22.6)	0.112
Atrial fibrillation	303 (35.0)	44 (34.9)	115 (37.3)	144 (33.3)	0.517
Pulmonary edema	58 (6.7)	11 (8.7)	19 (6.2)	28 (6.5)	0.604
Syncope	91 (10.5)	20 (15.9)	27 (8.8)	44 (10.2)	0.086*
Permanent pacemaker	79 (9.1)	15 (11.9)	20 (6.5)	44 (10.2)	0.116
Previous BAV	13 (1.5)	4 (3.2)	4 (1.3)	5 (1.2)	0.243
Previous PCI	339 (39.1)	58 (46.0)	119 (38.6)	162 (37.4)	0.214
Previous CABG	196 (22.6)	33 (26.2)	92 (29.9)	71 (16.4)	<0.001^†^
Previous SAVR	35 (4.2)	9 (7.1)	12 (3.9)	15 (3.5)	0.183
Previous cardiac surgery	219 (25.3)	36 (28.6)	101 (32.8)	82 (18.9)	<0.001^†^
Pulmonary hypertension^c^	(*N* = 848) 150 (17.7)	47 (37.3)	(*N* = 307) 55 (17.9)	(*N* = 415) 48 (11.6)	<0.001*^,^^†^

Values are median (IQR) or *n* (%), unless otherwise noted.

NYHA, New York Heart Association; EuroSCORE, European System for Cardiac Operative Risk Evaluation; LVEF, left ventricular ejection fraction; eGFR, estimated glomerular filtration rate; CAD, coronary artery disease; MI, myocardial infarction; PAD, peripheral artery disease; COPD, chronic obstructive pulmonary disease; BAV, balloon valvuloplasty; PCI, percutaneous coronary intervention; CABG, coronary artery by-pass graft.

* Denotes statistically significance on pairwise comparison between period 1 and period 2.

^†^ Denotes statistically significance on pairwise comparison between period 2 and period 3.

^a^ Defined as diagnosis set by primary care physician and/or taking hypertensive reducing medication.

^b^ Defined as both having had cancer or active cancer.

^c^ Defined as having a systolic pulmonary artery pressure (SPAP) > 55 mmHg.

### 3.2 Periprocedural, early, and late mortality by period

**Figure 1:**
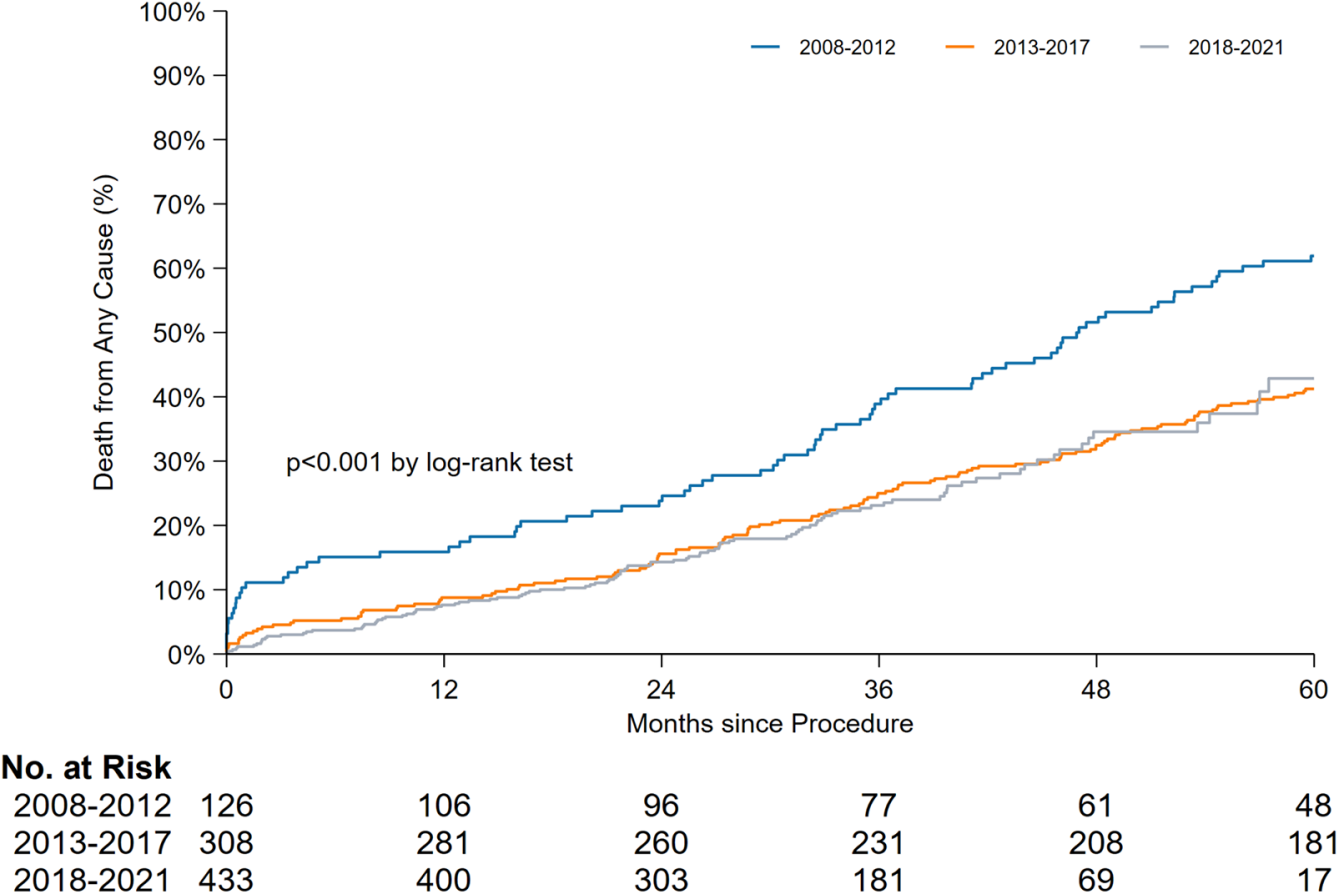
Time-to-event curves for late mortality between periods 1, 2 and 3.

**Figure 2:**
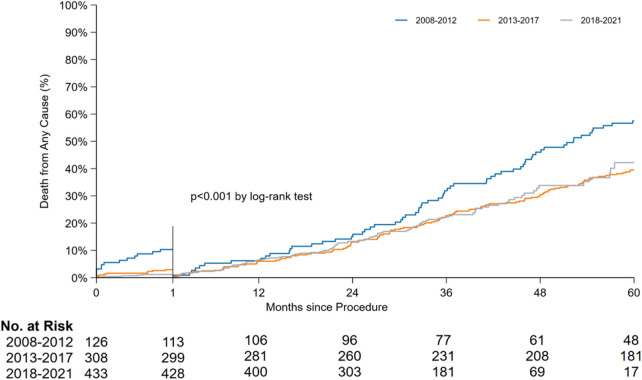
Landmark analysis after periprocedural mortality between periods 1, 2 and 3.

The periprocedural, early, and late mortality rates are shown in Figure 1. The periprocedural mortality rate significantly reduced from period 1 to period 2 (10.3% to 2.9% to 1.2%, *P* < 0.001). A pairwise comparison between period 2 and period 3 for periprocedural mortality (odds ratio [OR]: 0.39, 95% confidence interval [CI]: 0.13–1.17) and early mortality rates (OR: 1.11, 95% CI: 0.61–2.05) yielded no significant difference. Early mortality remained unchanged throughout the study period (5.6% to 5.8% to 6.5%, *P* = 0.904). Owing to the improvement in periprocedural mortality, the late mortality rate also improved. A landmark analysis of late mortality with the exclusion of periprocedural mortality using a log-rank test is presented in Figure 2, and the results are significant (*P* < 0.001).

### 3.3 Late mortality by sex and age groups

**Figure 3:**
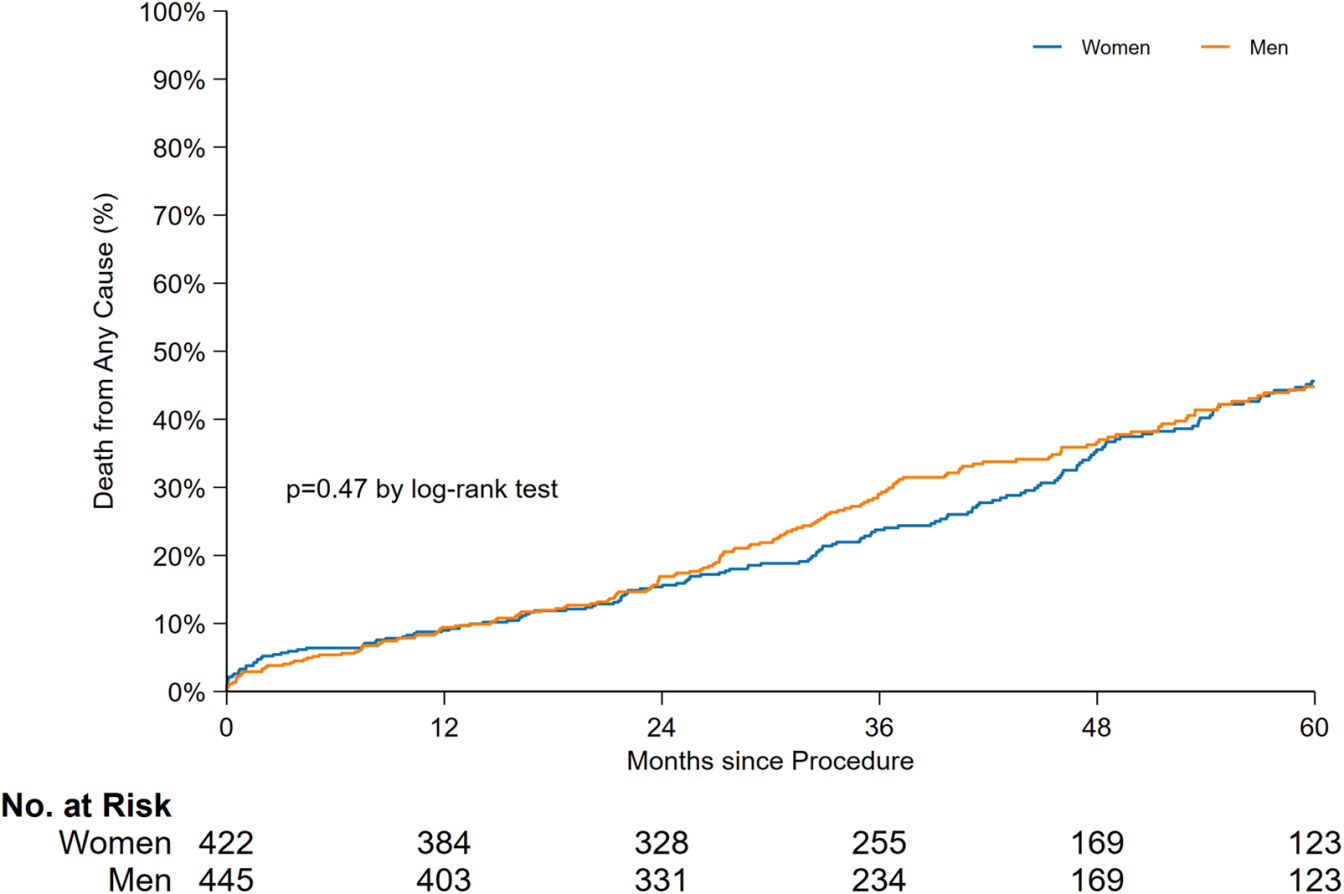
Time-to-event curves for late mortality stratified by sex.

A comparison of the late mortality rate between sexes is shown in Figure 3. Of the 867 patients, 422 women and 445 men were included in the time-to-event comparison, and no significant difference was found in the 5-year overall mortality rate. The median survival in years along with the 95% CI were as follows: 5.3 years [4.8–5.8] for women and 5.5 years [4.9–6.1] for men (*P* = 0.47).

**Figure 4:**
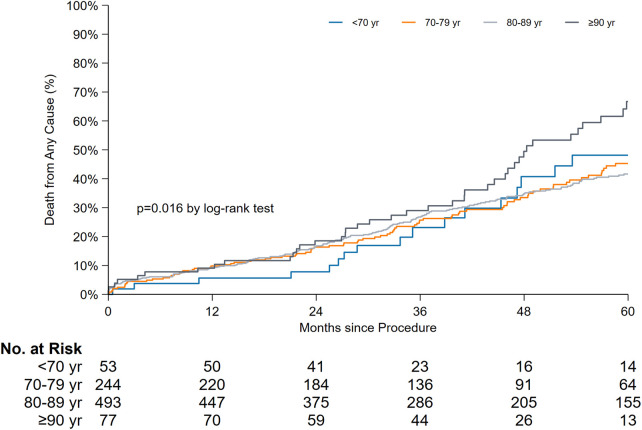
Time-to-event curves for late mortality stratified by age groups.

In terms of age-stratified late mortality comparison (Figure 4), a significant difference was observed. The median survival rates in years along with the 95% CI for each age group were as follows: < 70 years, 5.3 years [3.2–7.4]; 70–79 years, 5.5 years [4.6–6.4]; 80–89 years, 5.6 years [5.2–6.0]; and ≥90 years, 4.0 years [3.3–4.6] (*P* = 0.016). When the overall survival was compared with the exclusion of patients aged ≥90 years, the log-rank test results were not significant (*P* = 0.81).

### 3.4 Procedural and VARC-3 complications

The procedural and VARC-3 complications are listed in Table 2 and Table 3. A significant reduction was observed in the prevalence of major access-related nonvascular complications and VARC-3 type 2–3 bleeding in period 1 compared with that in periods 2 and 3. The rate of early PPI increased between periods 1 and 2, and the PPI rate continued to increase from periods 2 to 3. An inverse relationship was observed between PPI and PVL, as a high proportion of patients had none/trace PVL in later periods. Percutaneous closure device failure was only observed in period 3 owing to the implementation of percutaneous TF-TAVI.

**Table 2:** Post-procedural complications.

	All patients (*N* = 867)	2008–2012 (*N* = 126)	2013–2017 (*N* = 308)	2018–2021 (*N* = 433)	*P*-value
Myocardial infarction	8 (0.9)	4 (3.2)	3 (1.0)	1 (0.2)	0.01
Stroke	20 (2.3)	2 (1.6)	7 (2.3)	11 (2.5)	0.82
Kidney failure	15 (1.7)	4 (3.2)	1 (0.3)	10 (2.3)	0.05*^,^^†^
Respiratory distress syndrome	16 (1.8)	4 (3.2)	5 (1.6)	7 (1.6)	0.487
Infection	47 (5.4)	9 (7.1)	17 (5.5)	21 (4.8)	0.604
Sepsis	5 (0.6)	1 (0.8)	3 (1.0)	1 (0.2)	0.396
Tamponade	24 (2.8)	3 (2.4)	10 (3.2)	11 (2.5)	0.812
Intra-aortic balloon pump	11 (1.3)	10 (7.9)	1 (0.3)	0 (0.0)	<0.001*
Rupture of annulus	5 (0.6)	1 (0.8)	4 (1.3)	0 (0.0)	0.067
Paravalvular leak ≥2^a^	76/849 (8.8)	18/99 (15.4)	32/271 (10.6)	26/403 (6.1)	0.004^†^
Any re-operation	34 (3.9)	4 (3.2)	16 (5.2)	14 (3.2)	0.358
Any-cause rehospitalization	86 (9.9)	11 (8.7)	28 (9.1)	47 (10.9)	0.651
Valve embolization	5 (0.5)	4 (3.2)	0.0	1 (0.2)	<0.001*
Mors in tabula	6 (0.7)	3 (2.4)	2 (0.6)	1 (0.2)	0.037

Values are *n* (%), unless otherwise noted.

* Denotes statistically significance on pairwise comparison between period 1 and period 2.

^†^ Denotes statistically significance on pairwise comparison between period 2 and period 3.

^a^ Indicates a paravalvular leak of moderate or higher severity.

**Table 3:** Valve academic research consortium-3 defined complications.

	All patients (*N* = 867)	2008–2012 (*N* = 126)	2013–2017 (*N* = 308)	2018–2021 (*N* = 433)	*P*-value
Permanent pacemaker <30 days after procedure^a^	71 (9.0)	1 (0.9)	20 (6.9)	50 (12.9)	<0.001*^,^^†^
Permanent pacemaker >30 days after procedure^a^	15 (1.9)	4 (3.6)	6 (2.1)	5 (1.3)	0.29
Major vascular complication	17 (2.0)	5 (4.0)	6 (1.9)	6 (1.4)	0.184
Minor vascular complication	50 (5.8)	1 (0.8)	16 (5.2)	33 (7.6)	0.013*
Percutaneous closure-device failure^b^	14 (1.6)	N/A	N/A	14 (3.2)	N/A
Major cardiac structural complication	22 (2.5)	4 (3.2)	9 (2.9)	9 (2.1)	0.684
Minor cardiac structural complication	2 (0.2)	1 (0.8)	0 (0.0)	1 (0.2)	0.294
Major access-related non-vascular complication	7 (0.8)	5 (4.0)	1 (0.3)	1 (0.2)	<0.001*
Minor access-related non-vascular complication	1 (0.1)	0 (0.0)	1 (0.3)	0 (0.0)	0.403
Conversion to open surgery during procedure	15 (1.7)	4 (3.2)	6 (1.9)	5 (1.2)	0.290
Conversion to open surgery <30 days after procedure	14 (1.6)	2 (1.6)	7 (2.3)	5 (1.2)	0.492
Unplanned use of mechanical circulatory support	12 (1.4)	6 (4.8)	5 (1.6)	1 (0.2)	<0.001^†^
VARC-3 bleeding and transfusions					
Type 1 bleeding	68 (7.8)	13 (10.3)	22 (7.1)	33 (7.6)	<0.001
Type 2 bleeding	96 (11.1)	42 (33.3)	34 (11.0)	20 (4.6)	<0.001*^,^^†^
Type 3 bleeding	29 (3.3)	15 (11.9)	9 (2.9)	5 (1.2)	<0.001*
Prosthetic aortic valve regurgitation^c^					
None/trace	453 (53.4)	42 (35.9)	147 (48.5)	264 (61.5)	<0.001^*,†^
Mild	320 (37.7)	57 (48.7)	124 (40.9)	139 (32.4)	0.002^†^
Moderate	72 (8.5)	16 (13.7)	31 (10.2)	25 (5.8)	0.01^†^
Severe	4 (0.5)	2 (1.7)	1 (0.3)	1 (0.2)	0.107

Values are *n* (%), unless otherwise noted.

N/A, not applicable.

* Denotes statistically significance on pairwise comparison between period 1 and period 2.

^†^ Denotes statistically significance on pairwise comparison between period 2 and period 3.

^a^ Patients with prior pacemakers were excluded in the analysis.

^b^ Defined as failure of the device to achieve hemostasis as intended.

^c^ Data missing for 9 patients in period 1, 5 patients in period 2 and 4 patients in period 3.

### 3.5 Procedural information

Table 4 presents the procedural information and valve characteristics. The implementation rate of TF-TAVI increased throughout the study period, and TF-TAVI accounted for 94.2% of all procedures performed in period 3. TA-TAVI, which accounted for almost half of the procedures performed in period 1, was rarely employed in period 3. The rate of performing TSc-TAVI increased in period 3. Percutaneous access was introduced in period 3 and was employed in half of the procedures. Local anesthesia was increasingly used and accounted for 91.2% of all procedures performed in period 3. The median procedural time (from 90 min to 60 min) and length of hospital stay (from 8 days to 4 days) significantly improved.

**Table 4:** Procedural information and valve characteristics.

	All patients (*N* = 867)	2008–2012 (*N* = 126)	2013–2017 (*N* = 308)	2018–2021 (*N* = 433)	*P*-Value
Trans-femoral access (TF-TAVI)	739 (85.2)	69 (54.8)	262 (85.1)	408 (94.2)	<0.001*^,^^†^
Trans-apical access (TA-TAVI)	96 (11.1)	56 (44.4)	33 (10.7)	7 (1.6)	<0.001*^,^^†^
Trans-aortic access (TAo-TAVI)	12 (1.4)	1 (0.8)	10 (3.2)	1 (0.2)	0.002^†^
Trans-subclavian/axillary access (TSc-TAVI)	16 (1.8)	0 (0.0)	3 (1.0)	13 (3.0)	0.032
Trans-carotid access (TC-TAVI)	4 (0.5)	0 (0.0)	0 (0.0)	4 (0.9)	0.133
Surgical cut-down	667 (76.9)	126 (100)	308 (100)	233 (53.8)	<0.001^†^
Percutaneous technique	200 (23.1)	0 (0)	0 (0)	200 (46.2)	<0.001^†^
Localized anesthesia	549 (63.3)	0 (0)	154 (50)	395 (91.2)	<0.001*^,^^†^
Duration of procedure, minutes	69 (55–90)	96 (85–114)	66 (55–83)	64 (51–80)	<0.001*
Length of stay, days	5 (4–7)	8 (7–11)	6 (4–8)	4 (3–5)	<0.001*^,^^†^
Post-operative gradient^a^	10 (8–13)	11 (9–14)	10 (8–13)	10 (8–13)	0.752
Success^b^	849 (97.9)	122 (96.8)	302 (98.1)	425 (98.2)	0.643
Valve type: ballon-expanding					
Edwards SAPIEN	54 (6.2)	54 (42.9)	0	0	<0.001
Edwards SAPIEN XT	116 (13.4)	63 (50.0)	53 (17.2)	0	
Edwards SAPIEN 3	327 (37.7)	0	231 (75.0)	96 (22.2)	
Edwards SAPIEN 3 Ultra	263 (30.3)	0	0	263 (60.7)	
JenaValve	1 (0.1)	0	1 (0.3)	0	
Valve type: Self-expanding					
Medtronic CoreValve	9 (1.0)	9 (7.1)	0	0	
Portico	14 (1.6)	0	14 (4.5)	0	
Medtronic CoreValve Evolut R	40 (4.6)	0	9 (2.9)	31 (7.2)	
Medtronic CoreValve Evolut Pro	42 (4.8)	0	0	42 (9.7)	
Medtronic CoreValve Evolut PRO +** **	1 (0.1)	0	0	1 (0.2)	

Values are median (IQR) or *n* (%), unless otherwise noted.

* Denotes statistically significance on pairwise comparison between period 1 and period 2.

^†^ Denotes statistically significance on pairwise comparison between period 2 and period 3.

^a^ Data missing for 12 patients in period 1, 11 patients in period 2 and 4 patients in period 3.

^b^ Defined as deployment of the valve in its intended position.

### 3.6 Multivariable regression analysis of early mortality

The results of the multivariable regression analysis are shown in Table 5. The variables included in the full model were selected via comparison of the 53 patients who died >30 to ≤365 days to the 787 survivors past 365 days. The 27 patients who died within the first 30 days were excluded (full analysis is available in Supplementary Material). We identified increasing EuroSCORE II, systemic corticosteroid treatment (SCT), post-operative infection, post-operative kidney failure and any-cause rehospitalization as independent predictors for early mortality.

**Table 5:** Results of multivariable regression analysis of early mortality (>30 to ≤365 days).

	Unadjusted			Adjusted^a^		
Predictor^b^	OR	95% CI	*P*-value	OR	95% CI	*P*-value
EuroSCORE II	1.07	1.04–1.10	<0.001	1.07	1.04–1.10	<0.001
Systemic corticosteroid treatment	2.40	1.27–4.53	0.007	2.35	1.24–4.48	0.009
Post-operative infection	3.40	1.44–8.04	0.005	3.31	1.38–7.89	0.007
Post-operative kidney failure	4.49	1.06–19.10	0.042	4.53	1.06–19.43	0.042
Any-cause rehospitalization	2.44	1.16–5.13	0.019	2.42	1.14–5.13	0.021

Variables removed due to multicollinearity: Previous cardiac surgery, moderate PVL.

VARC-3, Valve academic research consortium-3; COPD, chronic pulmonary obstructive disease; RDS, respiratory distress syndrome; CABG, coronary artery by-pass graft; BAV, balloon valvuloplasty; eGFR, estimated glomerular filtration rate; PAD, peripheral artery disease; PVL ≥2, paravalvular leak moderate or higher; LVEF, left ventricular ejection fraction.

^a^ Model adjusted for age and sex.

^b^ Stepwise removal of variables from full model: VARC-3 type 3 bleed, COPD, VARC-3 type 2 bleed, pre-operative gradient, post-operative RDS, pulmonary edema, post-operative stroke, previous CABG, pulmonary hypertension, previous BAV, eGFR, PAD, PVL ≥2, BMI, any re-operation, pre-operative LVEF, post-operative sepsis.

## 4 Discussion

The main results of our study were as follows: (1) Periprocedural mortality declined, but early mortality remained stable over time. Due to improvements in periprocedural mortality, late mortality has also improved. (2) No significant difference was observed in late mortality stratified by sex. In relation to the age-stratified analysis, no significant differences were observed in the <70-, 70–79-, and 80–89-year age groups. A significant difference was observed when nonagenarians were included. (3) In terms of complications, an inverse relationship was found between PPI and PVL, with increasing PPI and decreasing PVL. (4) In terms of patient characteristics and comorbidities, the prevalence of most comorbidities decreased, and the risk profile tended to towards healthier patients. However, the median age of most TAVI recipients remained relatively unchanged at approximately 82 years.

When comparing our mortality data to other real-world data, a study from Germany reported periprocedural mortality rates of 6.3% in period 2 and 4.3% in period 3 (21), and other TAVI registries had comparable results to ours (22, 23). Although most registries depict improvements in periprocedural mortality and 1-year mortality, Arnold et al. demonstrated that between 2012 and 2018, the most important factors contributing to short-term outcomes were device technology and procedural factors (24). A study conducted by our group also found that TA-TAVI was a predictor of periprocedural mortality (25), comprising almost half of the procedures in period 1. In contrast with periprocedural mortality, early mortality did not improve during the study period. Our multivariable regression analysis found SCT to be the strongest predictive pre-operative dichotomous variable of early mortality. However, without having the cause of death for these patients extrapolating causal relationship is difficult. Nevertheless, we believe this to be a surrogate for long-standing chronic illness, which neither the EuroSCORE II nor the 2023 STS ACSD calculators incorporate. These insights demonstrate that identifying patients for whom TAVI is futile remains a clinical challenge. Potential reasons for this might include the low precision of the available risk score calculators (26) or lackluster assessment of frailty (27, 28). Inclusion of frailty and chronic illnesses to risk calculators can potentially aide heart teams to better identify futility, and individual pre- and rehabilitation needs. As illustrated in the landmark analysis, past 30-days the survival rate was similar, and advanced age and increased disease severity likely contributed to the higher rate of late mortality in period 1. With an unchanged early mortality rate, the biggest contributors to the improved late mortality rate are assumed to be factors that affect periprocedural mortality. However, our study's definitions of mortality were introduced by VARC-3 in 2021, making it difficult to provide direct comparisons. As TAVI has emerged as the leading modality for treating AS, a TAVI-specific risk assessment tool should be developed in future large international multicenter studies (26, 27, 29).

At our center, the late mortality was similar between sexes and age groups, excluding patients aged >90 years, throughout the study period. Although this was an unadjusted analysis, the findings imply that the comorbidities have more significant contributions to survival than the chronological age, which is supported by the existing literature (30–32). A recent study by Masiero et al. investigating sex-specific considerations in TAVI found that female-specific anatomical and pathophysiological factors require a tailored approach to minimize periprocedural risks and improve postoperative outcomes (33). With the aging of the population and the increasing application of TAVI to younger patients who generally have more comorbidities, age should not be used as a primary criterion for deciding the type of treatment in patients with AS. Instead, anatomical, and procedural factors, the burden of comorbidities, and outcome expectations should guide the shared decision-making between the patient and the heart-team.

An inverse relationship was observed between the rates of PPI and PVL after TAVI. First, the reduction in the PVL rate can be attributed to the improved diagnostic workup using multidetector CT (MDCT) rather than using TEE to reduce the prosthesis/annulus sizing mismatch (34–36). Second, technical improvements in the latest generations of THVs with outer-sealing skirts have reduced the incidence of more than moderate PVL to less than 1% (8, 11). Furthermore, procedural improvements owing to enhanced operator expertise have led to more favorable THV deployments. In relation to the increasing rates of PPI, De Torres-Alba et al. found that the 3rd generation SAPIEN 3, with its increased strut height and outer sealing skirt, led to deeper implantations than the 2nd generation SAPIEN XT (37). As 3rd and 4th generation THV exerts more pressure on the membranous septum length, it may explain the increased risk of conduction disturbances (38). Considering this, the rates of PPI for 3rd generation and 4th generation (SAPIEN 3 Ultra) BEV are similar (39), but our data shows an increase in PPI from period 2 to period 3. This finding implies there are procedural factors or changes in the patient population that could explain the increased PPI rate. Our current hypotheses to the increasing PPI rate from period 2 to period 3 are: First, the increasing use of SEVs might have contributed as SEVs are found to have higher rates of PPI than BEVs (38). Second, the possibility of slippage during the rapid pacing during BEV deployment, potentially due to the higher procedural volume (less time available per procedure) or impatience of operators in training. Third, operators facilitating for a potential valve-in-valve procedure leading to deeper placements to mitigate the risk of future coronary obstruction. In conclusion, identifying the causal explanation to period 3's increased PPI rate requires analysis of pre-operative arrhythmias, CT measurements of the membranous septum and its relation to the implantation depth (ID). As demonstrated by Sammour and colleagues, reducing the SAPIEN-3 ID by 50% led to an equal reduction in PPI and new onset left bundle branch block (40). Adopting techniques which reduce PPI, as well as improving valve design to minimize radial forces exerted onto the conduction system will be important to reduce TAVI morbidity and mortality.

A patient demographic that trends towards lower-risk patients, but with little or no change in the age comparable to the findings of similar studies (21–23, 41). This was expected, as TAVI was initially only for those deemed too high risk for SAVR. Currently, TAVI is preferred over SAVR in 75% of patients aged 65–80 years (42). These observations are supported by the results of the landmark TAVI vs. SAVR trials, which favor TAVI owing to its less invasive nature, shorter hospital stay, and quicker recovery (7–9). Meanwhile, care should be taken when interpreting the changes in comorbidities owing to the small sample in our study. However, the established risk factors for SAVR, such as previous cardiac surgery or CABG (42), have been markedly reduced, implying that more patients in the low-risk strata are being offered TAVI. Although the indications for TAVI are expanding to low-risk patients, this emphasizes the need to collaborate with a heart-team, determine the patient's preference, and establish a shared decision-making approach to develop a more tailored AS treatment.

## 5 Study limitations

The study has multiple limitations. First, the single-center retrospective observational design of the study might not be generalizable to other hospitals or populations. A multi-center study could provide more robust findings. The retrospective data-collection implies more missing data compared to a retrospective study design. Additionally, UNN is the regional hospital for a large geographic area comparable to the United Kingdom. Therefore, several unmeasured confounders due to remoteness are present, related to delayed diagnostics and variabilities in frequency and quality of follow-up care. Second, the relatively small sample size increases the margin of error in our predictive models, as is evident by the broad confidence intervals. Also, low statistical power and susceptibility of outliers are common problems related to small sample sizes. This is especially important with regards to our finding that early mortality has remained unchanged. These observations should be investigated in larger studies to both validate the results and properly identify areas of improvement. Third, there are several factors that limit the external validity of the study: The STS risk calculator was not used. There were no control groups, such as patients undergoing SAVR, to directly compare outcomes which could strengthen findings. Patients were selected for TAVI by the heart team based on multiple factors. This selection process could introduce bias that may impact results. However, throughout the study period there were few changes of the operators performing the procedures. That could imply that a larger part of the results and its interpretations are because of changes to device-technology and patient demographics contrary to variations of operator skills. Moreover, since our study is consistent with those of similar, larger registry-based observational studies, the volume of patients is sufficient to attain a high international standard. Lastly, our study is subject to data dredging, but adherence to the definitions and outcomes proposed by VARC-3 minimizes this risk.

## 6 Conclusion

As TAVI has revolutionized the treatment of severe AS in the last two decades, our findings indicate that most patients are octogenarians and that the burden of their comorbidities should be highly considered rather than their age when assessing for procedural outcomes. As most early complications of TAVI have been resolved, the rates of PPIs are high, and remains the Achilles' heel of TAVI. Important advancements in diagnostics, valve technology, and procedural techniques have improved the periprocedural mortality rates; however, early mortality remains unchanged and poses a clinical challenge that needs to be addressed in the future.

## Data Availability

The datasets presented in this article are not readily available because the data used in our study cannot be shared, even upon request. This is because they contain sensitive medical information which can compromise patient anonymity. Access to and the distribution of such data are strictly regulated by law. Requests to access the datasets should be directed to martin.p.hoydahl@uit.no.
