# Supplementary material for: Transcatheter aortic valve implantation (from inception to standard treatment): a single-center observational study
Source: Front Cardiovasc Med. 2024 Jan 15;11:1298346. doi: 10.3389/fcvm.2024.1298346 (PMC10822919; doi:10.3389/fcvm.2024.1298346)
Supplement: Supplementary file 1 [file Table1.docx]

Supplementary Material to Transcatheter Aortic Valve Implantation (from Inception to Standard Treatment): A Single-Center Observational Study

**Martin Petter Høydahl^1*^, Rolf Busund^1,2^, Assami Rösner^1,3^ and Didrik Kjønås^1,4^**

^1^Cardiovascular Research Group, Institute of Clinical Medicine, The Arctic University of Norway, Tromsø, Norway

^2^Department of Cardiothoracic Surgery, University Hospital of North Norway, Tromsø, Norway

^3^Department of Cardiology, University Hospital of North Norway, Tromsø, Norway

^4^Department of Gastrointestinal Surgery, University Hospital of North Norway, Tromsø, Norway

Content

[1 Table 1: Baseline Characteristics between Early Mortality and Survivors 1](#_Toc153977512)

[2 Table 2: Post-procedural Complications. 2](#_Toc153977513)

[3 Table 3: Valve Academic Research Consortium-3 Defined Complications. 2](#_Toc153977514)

[4 Table 4: Procedural Information 3](#_Toc153977515)

[5 Table 5: Valve sizes. 3](#_Toc153977516)

# Table 1: Baseline Characteristics between Early Mortality and Survivors

|  | All Patients  (N=840) | Early Mortality (N=53) | Survivors (N=787) | P Value |
| --- | --- | --- | --- | --- |
| Age, years | 82 (77 – 86) | 81 (76 – 86) | 82 (77 – 86) | 0.57 |
| Male sex | 432 (51.4) | 29 (54.7) | 403 (51.2) | 0.62 |
| NYHA class ≥3 | 743 (88.5) | 51 (96.2) | 692 (87.9) | 0.067 |
| EuroSCORE II | 5.0 (3.1 – 8.8) | 9.4 (5.7 – 19.9) | 4.9 (3.0 – 14.6) | <0.001 |
| Body-mass-index, kg/m^2^ | 26.2 (23.5 – 29.4) | 25.2 (22.8 – 28.3) | 26.2 (23.5 – 29.4) | 0.068 |
| Effective Orifice Area, cm^2^ | 0.6 (0.5 – 0.7) | 0.6 (0.5 – 0.7) | 0.6 (0.5 – 0.7) | 0.22 |
| Body Surface Area, m^2^ | 1.83 (1.67 – 1.97) | 1.80 (1.63 – 2.01) | 1.84 (1.68 – 1.96) | 0.32 |
| Pre-operative LVEF | 54 (42.0 – 60.0) | 48.5 (35.0 – 55.0) | 55 (45 – 60) | 0.008 |
| Pre-operative gradient | 51 (44.0 – 60.0) | 48 (41 – 55) | 51 (45 – 60) | 0.037 |
| eGFR | 63.7 (49.6 – 79.2) | 50.5 (35.1 – 69.3) | 64 (50.9 – 79.6) | <0.001 |
| Mitral valve regurgitation >2 | 66 (7.9) | 3 (5.7) | 63 (8.0) | 0.54 |
| Aortic valve regurgitation >2 | 89 (10.6) | 6 (11.3) | 83 (10.5) | 0.86 |
| Hypertension‡ | 520 (61.9) | 34 (64.2) | 486 (61.8) | 0.73 |
| Diabetes Mellitus | 182 (21.7) | 15 (28.3) | 167 (21.2) | 0.23 |
| CAD | 483 (57.5) | 34 (64.2) | 449 (57.1) | 0.31 |
| Previous MI | 242 (28.8) | 16 (30.2) | 226 (28.7) | 0.82 |
| Cerebrovascular disease | 207 (24.6) | 17 (32.1) | 190 (24.1) | 0.20 |
| Previous stroke | 125 (14.9) | 10 (18.9) | 115 (14.6) | 0.40 |
| PAD | 242 (28.8) | 24 (45.3) | 218 (27.7)) | 0.006 |
| COPD | 247 (29.4) | 20 (37.7) | 227 (28.8) | 0.17 |
| Systemic Corticosteroid Treatment | 151 (18.0) | 18 (34.0) | 133 (16.9) | 0.002 |
| Porcelain aorta | 20 (2.4) | 0 (0.0) | 20 (2.5) | 0.24 |
| Cancer§ | 179 (21.3) | 14 (26.4) | 165 (21.0) | 0.35 |
| Atrial fibrillation | 293 (34.9) | 21 (39.6) | 272 (34.6) | 0.45 |
| Pulmonary edema | 55 (6.5) | 7 (13.2) | 48 (6.1) | 0.043 |
| Syncope | 87 (10.4) | 3 (5.7) | 84 (10.7) | 0.25 |
| Permanent pacemaker | 78 (9.3) | 4 (7.5) | 74 (9.4) | 0.65 |
| Previous BAV | 11 (1.3) | 2 (3.8) | 9 (1.1) | 0.10 |
| Previous PCI | 324 (38.6) | 23 (43.4) | 301 (38.2) | 0.46 |
| Previous CABG | 191 (22.7) | 20 (37.7) | 171 (21.7) | 0.007 |
| Previous SAVR | 35 (4.2) | 2 (3.8) | 33 (4.2) | 0.88 |
| Previous cardiac surgery | 214 (25.5) | 21 (39.6) | 193 (24.5) | 0.015 |
| Pulmonary Hypertension‖ | 140/821 (17.1) | 16/51 (31.4) | 124/770 (16.1) | 0.005 |
| Values are median (IQR) or n (%), unless otherwise noted.  Abbreviations: New York Heart Association (NYHA), European System for Cardiac Operative Risk Evaluation (EuroSCORE), left ventricular ejection fraction (LVEF), estimated glomerular filtration rate (eGFR), coronary artery disease (CAD), myocardial infarction (MI), peripheral artery disease (PAD), chronic obstructive pulmonary disease (COPD), balloon valvuloplasty (BAV), percutaneous coronary intervention (PCI), coronary artery by-pass graft (CABG).  *Denotes statistically significance on pairwise comparison between period 1 and period 2.  †Denotes statistically significance on pairwise comparison between period 2 and period 3.  ‡Defined as diagnosis set by primary care physician and/or taking hypertensive reducing medication.  §Defined as both having had cancer or active cancer  ‖Defined as having a systolic pulmonary artery pressure (SPAP) >55mmHg. | | | | |

# Table 2: Post-procedural Complications.

|  | All Patients  (N=840) | Early Mortality (N=53) | Survivors (N=787) | P Value |
| --- | --- | --- | --- | --- |
| Myocardial infarction | 3 (0.4) | 0 (0.0) | 3 (0.4) | 0.82 |
| Stroke | 16 (1.9) | 3 (5.7) | 13 (1.7) | 0.039 |
| Kidney failure | 11 (1.3) | 3 (5.7) | 8 (1.0) | 0.004 |
| Respiratory distress | 9 (1.1) | 2 (3.8) | 7 (0.9) | 0.048 |
| Infection | 42 (5.0) | 9 (17.0) | 33 (4.2) | <0.001 |
| Sepsis | 3 (0.4) | 2 (3.8) | 1 (0.1) | 0.011 |
| Tamponade | 14 (1.7) | 1 (1.9) | 13 (1.7) | 0.90 |
| Intra-aortic balloon pump | 3 (0.4) | 0 (0.0) | 3 (0.4) | 0.82 |
| Rupture of annulus | 3 (0.4) | 0 (0.0) | 3 (0.4) | 0.82 |
| Paravalvular leak ≥2‡ | 73 (8.7) | 9 (17.0) | 64 (8.2) | 0.028 |
| Any re-operation | 35 (4.2) | 5 (9.4) | 30 (3.8) | 0.047 |
| Any-cause rehospitalization | 84 (10.0) | 12 (22.6) | 72 (9.1) | 0.002 |
| Valve embolization | 2 (0.2) | 0 | 2 (0.3) | 0.88 |
| Values are n (%), unless otherwise noted.  *Denotes statistically significance on pairwise comparison between period 1 and period 2  †Denotes statistically significance on pairwise comparison between period 2 and period 3  ‡Indicates a paravalvular leak of moderate or higher severity | | | | |

# Table 3: Valve Academic Research Consortium-3 Defined Complications.

|  | All Patients  (N=840) | Early Mortality (N=53) | Survivors (N=787) | P Value |
| --- | --- | --- | --- | --- |
| Permanent pacemaker <30 days after procedure‡ | 69 (8.2) | 7.5 (4) | 65 (8.3) | 0.86 |
| Permanent pacemaker >30 days after procedure‡ | 15 (1.9) | 1 (1.9) | 14 (1.8) | 0.95 |
| Major vascular complication | 12 (1.4) | 1 (1.9) | 11 (1.4) | 0.77 |
| Minor vascular complication | 50 (6.0) | 3 (5.7) | 47 (6.0) | 0.93 |
| Percutaneous closure-device failure§ | 14 (1.7) | 0 (0.0) | 14 (1.8) | 0.34 |
| Major cardiac structural complication | 15 (1.8) | 1 (1.9) | 14 (1.8) | 0.95 |
| Minor cardiac structural complication | 2 (0.2) | 1 (1.9) | 1 (0.1) | 0.12 |
| Major access-related non-vascular complication | 6 (0.7) | 2 (3.8) | 4 (0.5) | 0.006 |
| Minor access-related non-vascular complication | 1 (0.1) | 0 (0.0) | 1 (0.1) | 0.94 |
| Conversion to open surgery during procedure | 8 (1.0) | 0 (0.0) | 8 (1.0) | 0.46 |
| Conversion to open surgery <30 days after procedure | 10 (1.2) | 0 (0.0) | 10 (1.3) | 0.41 |
| Unplanned use of mechanical circulatory support | 7 (0.8) | 1 (1.9) | 6 (0.8) | 0.38 |
| ◌ Type 1 bleeding | 64 (7.6) | 4 (7.5) | 60 (7.6) | 0.98 |
| ◌ Type 2 bleeding | 84 (10.0) | 10 (18.9) | 74 (9.4) | 0.026 |
| ◌ Type 3 bleeding | 23 (2.7) | 3 (5.7) | 20 (2.5) | 0.18 |
| ◌ PVL None/trace | 447 (53.5) | 26 (49.1) | 421 (53.8) | 0.50 |
| ◌ PVL Mild | 315 (37.7) | 18 (34.0) | 297 (38.0) | 0.56 |
| ◌ PVL Moderate | 69 (8.3) | 8 (15.1) | 61 (7.8) | 0.062 |
| ◌ PVL Severe | 4 (0.5) | 1 (1.9) | 3 (0.4) | 0.23 |
| Values are n (%), unless otherwise noted.  Abbreviations: Not applicable (N/A), Paravalvular leak (PVL)  *Denotes statistically significance on pairwise comparison between period 1 and period 2.  †Denotes statistically significance on pairwise comparison between period 2 and period 3.  ‡Patients with prior pacemakers were excluded in the analysis.  §Defined as failure of the device to achieve hemostasis as intended.  ‖Data missing for 9 patients in period 1, 5 patients in period 2 and 4 patients in period 3. | | | | |

# Table 4: Procedural Information

|  | All Patients  (N=840) | Early Mortality (N=53) | Survivors (N=787) | P Value |
| --- | --- | --- | --- | --- |
| Trans-femoral access (TF-TAVI) | 726 (86.4) | 43 (81.1) | 683 (86.8) | 0.25 |
| Trans-apical access (TA-TAVI) | 87 (10.4) | 8 (15.1) | 79 (10.0) | 0.24 |
| Trans-aortic access (TAo-TAVI) | 8 (1.0) | 0 (0.0) | 8 (1.0) | 0.46 |
| Trans-subclavian/axillary access (TSc-TAVI) | 15 (1.8) | 2 (3.8) | 13 (1.7) | 0.26 |
| Trans-carotid access (TC-TAVI) | 4 (0.5) | 0 (0.0) | 4 (0.5) | 0.77 |
| Surgical cut-down | 641 (76.3) | 49 (92.5) | 592 (75.2) | 0.004 |
| Localized anesthesia | 544 (64.8) | 35 (66.0) | 509 (64.7) | 0.84 |
| Duration of procedure, minutes | 68 (55 – 89) | 82 (65 – 99) | 67 (55 – 88) | 0.023 |
| Length of Stay, days | 5 (4 – 7) | 6 (4 – 9) | 4 (4 – 7) | 0.030 |
| Success‡ | 830 (98.8) | 53 (100) | 777 (98.7) | 0.41 |

# Table 5: Valve sizes.

| **Period 1 (2008 – 2012)** | | | |
| --- | --- | --- | --- |
|  | **All Patients (N=126)** | **Balloon-Expanding (N=117)** | **Self-Expanding (N=9)** |
| Edwards SAPIEN | 54 (42.9) | 54 (46.2) |  |
| ◌ 23mm | 29 (23.0) | 29 (24.8) |  |
| ◌ 26mm | 25 (19.8) | 25 (21.4) |  |
| Medtronic CoreValve | 9 (7.1) |  | 9 (100) |
| ◌ 26mm | 7 (7.1) |  | 7 (77.8) |
| ◌ 29mm | 1 (0.8) |  | 1 (11.1) |
| ◌ 31mm | 1 (0.8) |  | 1 (11.1) |
| Edwards SAPIEN XT | 63 (50.0) | 63 (53.8) |  |
| ◌ 23mm | 20 (15.9) | 20 (17.1) |  |
| ◌ 26mm | 31 (24.6) | 31 (26.5) |  |
| ◌ 29mm | 12 (9.5) | 12 (10.3) |  |
| **Period 2 (2013 – 2017)** | | | |
|  | **All Patients (N=308)** | **Balloon-Expanding (N=285)** | **Self-Expanding (N=23)** |
| Edwards SAPIEN XT | 53 (17.2) |  |  |
| ◌ 23mm | 14 (4.5) | 14 (4.9) |  |
| ◌ 26mm | 26 (8.4) | 26 (9.1) |  |
| ◌ 29mm | 13 (4.2) | 13 (4.6) |  |
| JenaValve | 1 (0.3) |  |  |
| ◌ 25mm | 1 (0.3) | 1 (0.4) |  |
| Edwards SAPIEN 3 | 231 (75) |  |  |
| ◌ 23mm | 70 (22.7) | 70 (24.6) |  |
| ◌ 26mm | 102 (33.1) | 102 (35.8) |  |
| ◌ 29mm | 59 (19.2) | 59 (20.7) |  |
| Portico | 14 (4.5) |  | 14 (60.9) |
| ◌ 23mm | 2 (0.6) |  | 2 (8.7) |
| ◌ 25mm | 5 (1.6) |  | 5 (21.7) |
| ◌ 27mm | 4 (1.3) |  | 4 (17.4) |
| ◌ 29mm | 3 (1.0) |  | 3 (13.0) |
| Medtronic CoreValve Evolut R | 9 (2.9) |  | 9 (37.5) |
| ◌ 23mm | 4 (1.3) |  | 4 (17.4) |
| ◌ 26mm | 2 (0.6) |  | 2 (8.7) |
| ◌ 29mm | 3 (1.0) |  | 3 (13.0) |
| **Period 3 (2018 – 2021)** | | | |
|  | **All Patients (N=433)** | **Balloon-Expanding (N=359)** | **Self-Expanding (N=74)** |
| Edwards SAPIEN 3 | 96 (22.2) | 96 (26.7) |  |
| ◌ 20mm | 1 (0.2) | 1 (0.3) |  |
| ◌ 23mm | 31 (7.2) | 31 (8.6) |  |
| ◌ 26mm | 40 (9.2) | 40 (11.1) |  |
| ◌ 29mm | 24 (5.5) | 24 (6.7) |  |
| Medtronic CoreValve Evolut R | 31 (7.2) |  | 31 (41.9) |
| ◌ 23mm | 3 (0.7) |  | 3 (4.1) |
| ◌ 26mm | 9 (2.1) |  | 9 (12.2) |
| ◌ 29mm | 12 (2.8) |  | 12 (16.2) |
| ◌ 34mm | 7 (1.6) |  | 7 (9.5) |
| Medtronic CoreValve Evolut Pro | 42 (9.7) |  | 42 (56.8) |
| ◌ 23mm | 4 (0.9) |  | 4 (5.4) |
| ◌ 26mm | 10 (2.3) |  | 10 (13.5) |
| ◌ 29mm | 18 (4.2) |  | 18 (24.3) |
| ◌ 34mm | 10 (0.9) |  | 10 (13.5) |
| Edwards SAPIEN 3 Ultra | 263 (60.7) | 263 (73.3) |  |
| ◌ 20mm | 4 (0.9) | 4 (1.1) |  |
| ◌ 23mm | 81 (18.7) | 81 (22.6) |  |
| ◌ 26mm | 122 (28.2) | 122 (34.0) |  |
| ◌ 29mm | 56 (12.9) | 56 (15.6) |  |
| Medtronic CoreValve Evolut PRO+ | 1 (0.2) |  | 1 (1.4) |
| ◌ 34mm | 1 (0.2) |  | 1 (1.4) |
| Values are n (%), unless otherwise noted. | | | |
